# Draft genome sequence of *Xylella fastidiosa* strain ATCC 35874 isolated from infected red oak in Washington, DC

**DOI:** 10.1128/MRA.00893-23

**Published:** 2023-12-01

**Authors:** Jonathan Shao, Wei Guan, Tingchang Zhao, Qi Huang

**Affiliations:** 1 U.S. Department of Agriculture, North East Area, Agricultural Research Service, Beltsville, Maryland, USA; 2 U.S. Department of Agriculture, Floral and Nursery Plants Research Unit, U.S. National Arboretum, Agricultural Research Service, Beltsville, Maryland, USA; 3 State Key Laboratory for Biology of Plant Diseases and Insect Pests, Institute of Plant Protection, Chinese Academy of Agricultural Sciences, Beijing, China; The University of Arizona, Tucson, Arizona, USA

**Keywords:** *Xylella fastidiosa*, red oak, bacterial leaf scorch, genome sequence

## Abstract

We report here the draft genome sequence of *Xylella fastidiosa* strain ATCC 35874. The strain was originally isolated from infected red oak in Washington, DC, and obtained from the American Type Culture Collection. The ATCC 35874 genome contains 2,543,332 bp and has a G + C content of 51.72%.

## ANNOUNCEMENT

*Xylella fastidiosa* is an economically important plant pathogenic bacterium, infecting 679 plant species across 88 families in the Americas, Europe, the Middle East, and Asia (1). It causes bacterial leaf scorch and decline in important landscape trees, including elm, mulberry, sycamore, and oak. At present, more than 200 complete or draft genome sequences of *X. fastidiosa* strains have been deposited in GenBank, but genome sequences of landscape tree strains are significantly underrepresented with only five such genomes published (2 – 6), including two mulberry strains Mul-MD from Maryland (2) and MUL0034 from California (3), one red oak strain Griffin-1 from Georgia (4), one sycamore strain Sy-VA from Virginia (5), and one elm strain ATCC 35873 from Washington, DC (6). We therefore sequenced the genome of *X. fastidiosa* strain ATCC 35874 obtained from the American Type Culture Collection (ATCC). Based on the information published by Wells et al. (7) and obtained from ATCC’s website, strain ATCC 35874 was originally isolated from twigs of infected red oak in Washington, DC.

**Fig 1:**
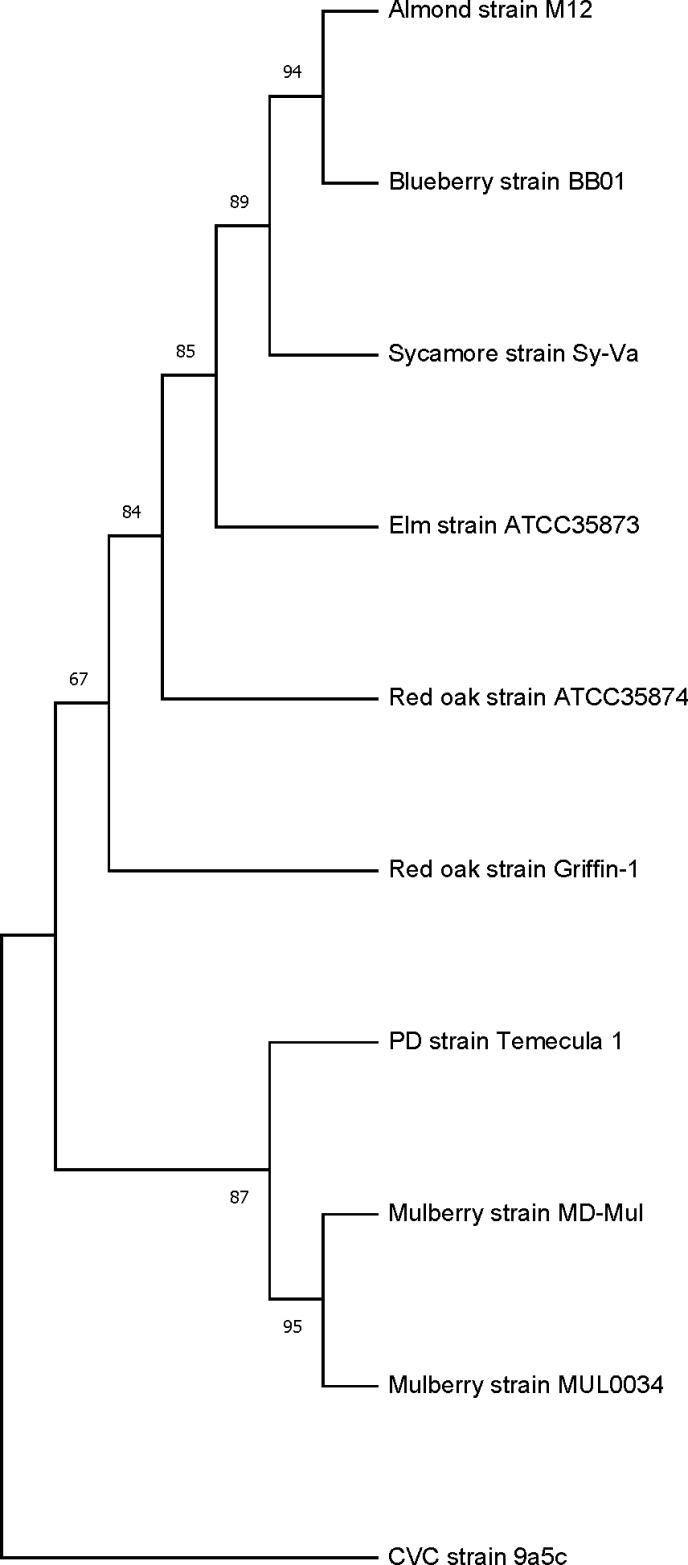
Phylogenetic relationship between the red oak strain ATCC 35874 and nine other strains of *Xylella fastidiosa* based on 16S rRNA gene sequences, showing that strain ATCC 35874 is more closely related to *X. fastidiosa* subsp. *multiplex* strains. The neighbor-joining phylogenetic tree was generated using MEGA11 (12). Bootstrap values (5,000 replicates) are represented at the nodes of the branches.

*X. fastidiosa* strain ATCC 35874 obtained from ATCC was streaked on periwinkle wilt (PW) medium plate (8) and grown at 28°C for 21 days. A single colony was picked and grown in PW liquid medium at 28°C for 14 days with gentle shaking. The bacterial cells were collected by centrifugation before genomic DNA was extracted using Qiagen’s Blood and Tissue kit (Qiagen Inc., Valencia, CA) according to the manufacturer’s instructions. Random shotgun and 3 kb mate-pair libraries of *X. fastidiosa* strain ATCC 35874 were generated and sequenced using Roche 454 GS (FLX titanium) pyrosequencing, resulting in 906,579 total reads with 128,698 shotgun reads and 777,881 mate-pairs—totaling 247,372,122 bases. The N50 is 682 for raw_Oak_5.GAC.454Reads (single end) and 487 for 6.TCA.454 (paired reads). After processing by the Newbler gsAssembler v2.7, the total number of reads from all libraries was 892,669 aligned reads, with 245,692,850 bases aligned. Read quality had a Q40plus bases at 99.97%. The genome was assembled into 218 contigs using the Newbler Assembler. The average length of all the contigs was 11,666 bases. The largest contig was 200,471 bases. The N50 contig size was 96,874 bases. All the contigs greater than 200 bases were run through an annotation pipeline using Prodigal (Prokaryotic Dynamic Programming Genefinding Algorithm) 2.6.3 (9) to predict coding regions. A total of 2,483 protein-encoding regions or putative genes were predicted. The translated protein sequences were annotated using the BLASTP program against the nr (non-redundant protein database) database with an e value cutoff of 0.001. Predictions of regions encoding 45 tRNAs and 6 rRNAs were made using tRNAscan-SE version 2.012 (10, 11) and barrnap version 0.9 programs, respectively. Phylogenetic relationship between strain ATCC 35874 and nine other *X. fastidiosa* strains is presented in Fig. 1. Default parameters were used for all software unless otherwise specified.

The depth of sequencing read coverage per base position was 65× for this draft genome of the *X. fastidiosa* strain ATCC 35874; the genome contains 2,543,332 bp and has a GC content of 51.72%.

## Data Availability

This Whole Genome Shotgun project has been deposited at DDBJ/ENA/GenBank under the accession JAVLTP000000000. The version described in this paper is version JAVLTP010000000. The raw sequences have been deposited in the NCBI SRA database under the accession numbers SRR25686646, SRR25686647, and SRR25686648.
